# Supplementary material for: Patterns of Intron Gain and Loss in Fungi
Source: PLoS Biol. 2004 Nov 30;2(12):e422. doi: 10.1371/journal.pbio.0020422 (PMC532390; doi:10.1371/journal.pbio.0020422)
Supplement: Table S1 — Also available at http://genes.mit.edu/NielsenEtAl/. (4.3 MB ZIP). [file pbio.0020422.st001.zip › NielsenEtAl/html/1289.html]

AN4913.1.NCU05151.1.MG09116.1.FG06553.1


```
 CLUSTAL W (1.82) Multiple Sequence Alignments - Introns Inserted


Sequence 1: NCU05151.1	815 aa
Sequence 2: MG09116.1	810 aa
Sequence 3: FG06553.1	805 aa
Sequence 4: AN4913.1	774 aa
Alignment Length: 820 aa
Number Identitical Residues: 412 aa
Alignment Score (without introns) 20777


MG09116.1 	MPAPEITEPNPPPLPSNIPDSASELAVRKHLPVSKLSEDDLAGVKEFRRAACYIAAA~MI
NCU05151.1	MGGTQITIPNPPPLPSHLPDNVLDLAVQIER--KNLPDNVRESLRAFQRAGCYIAAS~MI
FG06553.1 	MPGEVIDRPNPAPLDSSLPGAVLDLAAKAPK--KQLDKKIAQSLNDFQHAACYIAGS1MI
AN4913.1  	MPGEVIERPNPAPKPSHVPDLVEKLIIPAQK--TKLEKSDCDALHKYRRAAAYIAA-~--
          	* .  *  ***.*  * :*. . .*        .:* ..   .:. :::*..***.    

MG09116.1 	FLRDNVLLADDLTVDHIKPRLLG~HWGTCPGLVLVWAHLNLLVRDHDLHMIHVIGPGHGA
NCU05151.1	FLRDNVLLQDKLQVSHIKPRLLG~HWGTCPGLILVWSHLNVLIRNHDLNMIYVIGPGHGA
FG06553.1 	FLRDNVLLEREIKTEDIKPRLLG1HWGTCPGIILVWSHLNLLIRNHDLEMIFVIGPGHGA
AN4913.1  	----------------------G1HWGTCPGLILVYSHLNYLIKKQNLDMLYVVGPGHGA
          	                      * *******::**::*** *::.::*.*:.*:******

MG09116.1 	PAALAALWLEGSLERFYPGEYDRSSEGLKNLITRFSVPGGFPS2HINAETPGAIHEGGEL
NCU05151.1	PAALASLWLEGSLERFYPGEYDRNATGLRNLITRFSVPGGFPS~HINAETPGAIHEGGEL
FG06553.1 	PGALASLWLEGSLERFFPQKYGVNKDGLRNLITGFSVPGGFPS2HINSETPGSIHEGGEL
AN4913.1  	PGLLASLWLEGSLGKFYP-QYTKDKEGLHNLISTFSTSAGLPS2HINAETPGAIHEGGEL
          	*. **:******* :*:* :*  .  **:***: **...*:** ***:****:*******

MG09116.1 	GYALSVSFGAVMDKPDLVVACVIGDGEAESGPTAT~AWHSIKYLDPAESGAVLPILHVNG
NCU05151.1	GYALSVSFGAVMDNPDLIVTCVVGDGEAETGPTAT~AWHAIKYIDPKESGAVIPILHVNG
FG06553.1 	GYSLAVSFGAVMDNPDLIVTCLVGDGEAESGPTAA2AWHSIKYIDPAESGAVIPILHVNG
AN4913.1  	GYALSVSFGAVMDNPDLIVTCVVGDGEAETGPTAT2SWHAIKYIDPAESGAVLPILHVNG
          	**:*:********:***:*:*::******:****: :**:***:** *****:*******

MG09116.1 	FKISERTIFGCMDDKELVTLFTGYGYQVRIVEELDDIDNDLHQALEWGLSEIRNIQKAAR
NCU05151.1	FKISERTIFGCMDDKEIACLFSGYGYQVRIVEDLENIDDDLQSSLEWALVEIKKIQKAAR
FG06553.1 	FKISERTIFGCMDNKELVSLFSGYGYQPIIVETIEEIDAELSGVLEWAVSEIKKIQNAAR
AN4913.1  	FKISERTIFGCMDNREIVCLFTGYGYQVRIVEDLEDIDNDLHSAMSWAVEEIRNIQKAAR
          	*************::*:. **:*****  *** :::** :*   :.*.: **::**:***

MG09116.1 	SGEKKPIVKPRWPMIIMRTPK~GWSGPKEVDGKVVEGSFRSHQVPLPKAGNDETQLELLR
NCU05151.1	E-DNKPIVKPRWPMIVLRTPK~GWTGPKEVEGQIIEGSFHSHQVPLPKAGSDEKQLQALD
FG06553.1 	D--GKPIVKPRWPMIVLRTPK~GWTGPKKVDGEFIEGSFRSHQIPVPGAGQDGEHVKILQ
AN4913.1  	S--GKPIMKPQWPMIVLRTPK0GWSGPKELHGQFIEGSFHSHQVPLPNAKKDDEELQALQ
          	.   ***:**:****::**** **:***::.*:.:****:***:*:* * .*  .:: * 

MG09116.1 	DWLASYKIGDLLEDGGKPIRRITDTMPRKDEQKLGQVKETYAPEPGLEAVDWKPYGVEKK
NCU05151.1	EWLSSYRISELLDDEGKPTEAITRVLP-KAEKRLGQLKITYDPYIPLKLIDWKQFGVQKG
FG06553.1 	DWLKTYGTDRLLKD-GKPAESITDIIPQKDEKRLGQLKKTYNPHQQLQLPDWKSFGVEKL
AN4913.1  	KWLSSYKPDELFTESGDVIDEILSIIP-SDDKKLGMRPEAYKTHLPPDLPDWRQFCVKKG
          	.** :*  . *: : *.    *   :* . :::**    :* .    .  **: : *:* 

MG09116.1 	TTASCMATTGSFLDAVFQANPNRIRLFSPDELESNKLHAILDHTQRNFQWDAYSRGNGGR
NCU05151.1	VDESCMKSAGKFLDKLFQENPKTLRLFSPDELESNKLNAILDHTQRDFQWDEFSRAHGGR
FG06553.1 	SENSCMKQVGNFLNQVIKENPKSFRIFSPDELESNKLSAVFENTGRNFQWDEFSRAQGGR
AN4913.1  	DQFSAMKAIGSFIDQVFVKNPHTVRLFSPDELESNKLSAALSHTGRNFQWDEFSNAKGGR
          	   *.*   *.*:: ::  **: .*:*********** * :.:* *:**** :*..:***

MG09116.1 	VIETLSEHQCQGFMQ~GYTLTGRTAIFPSYESFLGIVHTMMVQYSKFVKI~AKEVKWRGD
NCU05151.1	VIEVLSEHNCQGFMQ~GYTLTGRTALFPSYESFLGIVHTMMVQYSKFVKI~AREVPWRGD
FG06553.1 	VIEILSEHCCQGWMQ0GYTLTGRTALFPSYESFLGIIHTMMVQYSKFNKI~ARETNWRGD
AN4913.1  	VIEVLSEHLCQGFMQ~GYTLTGRTGIFPSYESFLGIIHTMMVQYAKFAKM0AKETAWHHD
          	*** **** ***:** ********.:**********:*******:** *: *:*. *: *

MG09116.1 	LSSINYIETSTWARQEHNGFSHQNPSFIGAVLNIKPDSARVYLPPDANCFLSTAHHCVKS
NCU05151.1	LASINYIETSTWARQEHNGFSHQNPSFIGAVLNLKAEAARVYLPPDANCFLSTVHHVMDS
FG06553.1 	LSSINYIETSTWARQEHNGFSHQNPSFIGSVLNLKAEAARVYLPPDANCFLSTIHHCLGS
AN4913.1  	VSSINYIETSTWARQEHNGFSHQNPSFIGAVLKLKPYAARVYLPPDANTFLTTLHHCLKS
          	::***************************:**::*. :********** **:* ** : *

MG09116.1 	KNYVNLMIGSKQPTRVYLSPDEAAEHCKAGAGIFHFASTPAENGGDKTKSTADPDVVLVG
NCU05151.1	KNKTNLIIGSKQPTAVYLSPAEAADHCRQGASIWHFASSSDPNS-SAGESVQDPDVVLVG
FG06553.1 	KNYVNLMIGSKQPTAVFLSPDEAADHCRKGASVWEFASTD---------SGKQPDVVIAG
AN4913.1  	KNYINLMVGSKQPTPVYLSPEEAESHCRAGASIFKFCSTDG---------GLRPDVVLVG
          	**  **::****** *:*** ** .**: **.::.*.*:              ****:.*

MG09116.1 	IGVEVTAEAVAAAELLLDLCPELKVRMINVTDLMILIPESRHPHSLSDDKFEELFTADKP
NCU05151.1	IGVEVTFEVIKAAELLRSIAPALKIRVVNVTDLMILIEESKHPHALAKAKFIEMFTADRP
FG06553.1 	IGVEVTFEVVKAAELLRALIPDLRVRVVNVTDLMVLAAESRHPHALSRADFLDMFTEDKA
AN4913.1  	IGVEVMFEVIKAAAILRERCPELRVRVVNVTDLFILENEGAHPHALKHEAFDNLFTEDRS
          	*****  *.: ** :*    * *::*::*****::*  *. ***:*    * ::** *:.

MG09116.1 	VLFNYHGYATELQGLLFGRPRTERMTVEGYREEGSTTTPFDMMLANCVSRFHLARRAIRA
NCU05151.1	VLFNYHGYPTELQGLLFGREKAERMDVEGYQEEGSTTTPFDMMLRNRVSRFDVASWALKR
FG06553.1 	ICFNYHGYAAELQGLLFGRPGLHRMTVEGYKEEGSTTTPFDMMLLNWVSRFDVAKRALKG
AN4913.1  	IHFNYHGYVNELQGLLFGRPRLDRATIKGYKEEGSTTTPFDMMLVNEVSRYHVAKAAVTG
          	: ******  *********   .*  ::**:************* * ***:.:*  *:  

MG09116.1 	ASSSSAVDKNKLDQVLKEIQAREDRVRKYIDENGKG~T----------
NCU05151.1	GAERNAEVKNDLEGLLGDVDRRVKEVREFIGNEGKD1PDDIYQLPKFE
FG06553.1 	AAETNDKIKAKLDKTLKKIDDRVEEVRRYIAENGKD1PEDLYDMPKF-
AN4913.1  	GARFNEKVKLRHQELCSEFDHNIAETRKYIMNNHQA1DGNWS------
          	.:  .   *   :    ..: .  ..*.:* :: :    .
```
